# Willingness to use long-acting injectable pre-exposure prophylaxis (LAI-PrEP) among black cisgender women in the Southern United States

**DOI:** 10.1371/journal.pone.0330698

**Published:** 2025-09-02

**Authors:** Sylvia Shangani, Jaquetta M. Reeves, Kristin Heron, Jessica M. Sales, Kenneth K. Mugwanya

**Affiliations:** 1 Department of Community Health Sciences, Boston University School of Public Health, Boston, Massachusetts, United States of America; 2 Department of Social and Behavioral Sciences, Yale School of Public Health, New Haven, Connecticut, United States of America; 3 College of Nursing and Health Innovation, The University of Texas at Arlington, Arlington, Texas, United States of America; 4 Department of Psychology, Old Dominion University, Norfolk, Virginia, United States of America; 5 Department of Behavioral, Social, and Health Education Sciences, Rollins School of Public Health, Emory University, Atlanta, Georgia, United States of America; 6 Departments of Epidemiology and Global Health, University of Washington, School of Public Health, Seattle, Washington, United States of America; Neurocrine Biosciences Inc, UNITED STATES OF AMERICA

## Abstract

**Background:**

Long-acting injectable pre-exposure prophylaxis (LAI-PrEP) for HIV prevention may improve adherence for those with concerns with daily pills. Limited data exist on LAI-PrEP acceptability among Black women in the U.S., a population vulnerable to HIV. We assessed willingness to use LAI-PrEP among Black women eligible for PrEP in the Southern U.S.

**Methods:**

We conducted a cross-sectional online survey of HIV-negative Black women from March to June 2022 in the U.S. South. Participants provided information on sociodemographic characteristics, HIV knowledge, PrEP awareness, and use, stigma, risk perception, medical mistrust, and healthcare access. Multivariate logistic regression models determined factors associated with willingness to use LAI-PrEP.

**Results:**

Of 491 women, the mean (SD) age was 40.1 (17.5), 53% of participants had a college degree or lower, 79% were single, and 80% resided in urban/suburban settings. Thirty-nine percent were aware of PrEP before the study and 36.7% of women were willing to use LAI-PrEP. In multivariate analyses, PrEP awareness [adjusted odds ratio (aOR=2.37, 95% CI 1.40, 3.73, p < 0.001), having a personal clinician (aOR=2.01, 95% CI 1.10, 3.68, p = 0.02), HIV worried (aOR=1.78, 95% CI 1.09, 2.89, p = 0.02), and medical trust (aOR=1.41, 95% CI 1.03, 1.93, p = 0.04) were statistically associated with willingness to use LAI-PrEP. However, the healthcare stereotype (beliefs that healthcare is biased) had lower odds of using LAI-PrEP (aOR=0.94, 95% CI 0.89, 0.99, p = 0.04).

**Conclusion:**

Black women at risk for HIV are more likely to consider injectable PrEP when they understand HIV risk factors, are aware of PrEP, have a clinician, and trust the medical care. Implementing client-centered care interventions could effectively address medical mistrust and enhance engagement in HIV prevention services among Black women.

## Introduction

Black women in the U.S. experience disparities in HIV incidence, with a 1 in 48 lifetime risk of acquiring HIV compared to 1 in 880 for white women [1]. In 2022, Black women accounted for 50% of new HIV infections in U.S. women [2], despite being only 13% of women in the U.S. This rate of new HIV diagnoses among Black women (19.2) is 10 times the rate among White women (1.9) and 3 times the rate among Latina women (5.5) [3]. HIV incidence disparities are even more significant in the U.S. South for Black women [2,3]. In the U.S. South, Black women accounted for more than half (67%) of new HIV infections among all women living in the South [3]. The prevention of new infections remains a critical public health priority, especially for Black communities [4,5]. A key goal of the National HIV/AIDS Strategy is to reduce new HIV transmissions in the U.S. by disseminating PrEP [4,5].

Pre-exposure prophylaxis (PrEP) is a highly effective HIV prevention strategy for people who are at a high risk of HIV infection [6–8]. Daily oral pills (Emtricitabine/tenofovir disoproxil fumarate [F/TDF] and Emtricitabine/tenofovir alafenamide [F/TAF]) as well as long-acting injectable cabotegravir are FDA-approved PrEP medications, including for use among women [9,10]. These medications are effective interventions that have the potential to help end the HIV epidemic when implemented at scale for HIV-vulnerable populations [6–8]. However, uptake of these effective strategies to prevent HIV remains suboptimal among Black women in the U.S. Key barriers to PrEP access for this group include lack of access to PrEP, racism [11], discrimination [12], and HIV stigma, especially in the U.S. South [12,13]. Additionally, concerns about efficacy, side effects, stigma, and challenges related to daily adherence have contributed to the limited uptake of oral PrEP among Black women [14,15]. Other studies have also shown that unhealthy alcohol use, including binge drinking, lowers engagement in the PrEP care continuum [16].

Long-acting injectable pre-exposure prophylaxis (LAI-PrEP) offers a promising alternative that may address some of the barriers to oral PrEP uptake and adherence among Black women in the Southern U.S. LAI-PrEP/cabotegravir was FDA-approved in December 2021 [9] and has shown high efficacy, safety, and superiority over available oral PrEP. LAI-PREP (cabotgravir) reduced the risk of HIV infection by 89% compared with daily oral PrEP in a cohort of cisgender women in sub-Saharan Africa [17]. Preliminary data also show that LAI-PrEP is well tolerated during and after pregnancy [18]. LAI-PrEP involves receiving injections of a long-acting antiretroviral medication, such as cabotegravir, every one to three months, offering sustained protection against HIV with fewer adherence requirements compared to daily oral PrEP [17]. By alleviating the burden of daily pill-taking and offering a discreet, convenient option for HIV prevention, LAI-PrEP has the potential to empower Black women to take control of their HIV prevention efforts and decrease their susceptibility to HIV.

Understanding Black women’s willingness to use LAI-PrEP in the Southern U.S. requires exploration of their attitudes, beliefs, and experiences related to HIV prevention, healthcare access, and medical trust. Historical legacies of medical exploitation and mistreatment, coupled with ongoing disparities in healthcare access and quality, may influence Black women’s perceptions of LAI-PrEP and willingness to use [19,20]. We assessed Black women’s willingness to use LAI-PrEP in the Southern United States, examining the factors influencing their decisions and preferences. The study aims to inform the development of HIV prevention interventions that are culturally responsive and gender-affirming, and that address the unique needs and priorities of Black women in the Southern U.S., by focusing on the voices and perspectives of Black women. Through community-engaged research and collaborative partnerships, we can advance equitable access to LAI-PrEP and promote health equity for Black women disproportionately affected by HIV in the Southern U.S..

## Materials and methods

### Study design and participants

This study was an online cross-sectional survey of 491 HIV-negative cisgender Black women collected between March and June 2022. Participants (1) were 18 years and above, (2) identify as Black or African American, (3) assigned female at birth, (4) identified as a woman or non-binary, (5) resided in the U.S. South [Alabama, Arkansas, Delaware, The District of Columbia, Florida, Georgia, Kentucky, Louisiana, Maryland, Mississippi, North Carolina, Oklahoma, South Carolina, Tennessee, Texas, Virginia, and West Virginia], (6) report at least one act of condomless sex with a sexual partner in the past 6 months, consistent with CDC’s guidelines for PrEP eligibility for women [21]; (7) are fluent in English, and (8) are willing and able to provide informed consent.

### Study procedures

Participants were recruited via Qualtrics’s online panels. These panels include individuals from across the U.S. who are interested in participating in research studies. The study team worked with the Qualtrics program coordinator to administer a survey to a targeted pool of Black women who met the eligibility criteria above. Recruitment was facilitated through Qualtrics via email, and the survey was exclusively available to Qualtrics panel participants. We created an anonymous survey that Qualtrics distributed to their panels, allowing interested individuals to access it by clicking on the survey link. On average, participants took approximately 25 minutes to complete the survey. Those with concerns about their safety or privacy could decline participation. An emergency exit button at the bottom of each page redirected participants to a blank Google page if clicked. Additionally, to enhance safety, the survey did not include a back button.

Most survey questions featured binary or Likert scale response options and were distributed across 15 pages. The survey was piloted with 15% of the total respondents before full deployment. Following the pilot, the Qualtrics program coordinator implemented a speeding check that automatically terminated participants who were not responding thoughtfully, with a cut-off time set at seven minutes. To prevent multiple entries from the same respondent, Qualtrics placed a cookie in the participant’s browser upon survey submission. This allowed Qualtrics to recognize individuals who clicked the survey link again and restrict their access. IP addresses were not used to track unique respondents, ensuring participant anonymity. A copy of the survey has been included as supplementary material.

All research procedures were approved by the Old Dominion University Institutional Review Board (#1866874−2). All participants provided written informed consent for study procedures. The consent included an overview of study procedures, potential risks, benefits, and confidentiality measures. Participants were compensated $30 for their time, although Qualtrics designated the exact compensation rate.

### Measures

#### Primary outcome.

The primary outcome was the willingness to use LAI-PrEP. Participants were asked two questions: “Would you take an injection once a month to protect yourself from getting HIV?” and “Would you take an injection once every two months to protect yourself from getting HIV?” Participants who answered ‘yes” to any of these questions were categorized as willing to use LAI-PrEP. Participants were also asked about oral PrEP awareness, which was measured by asking participants if they had ever heard of a daily pill that an HIV-negative person can take to prevent getting HIV prior to the survey. Participants who answered “yes” were coded positive for oral PrEP awareness. We did not define LAI-PrEP for participants because its availability and public awareness were still limited among this population at the time of conducting the survey.

### Independent variables

#### Demographic variables.

Demographic data included participant age in years, education level, income, marital status, and nativity.

#### HIV risk awareness.

HIV risk awareness was assessed using Oglesby and Alemagno’s shortened 10-item HIV knowledge scale, which has been validated in both general and key populations [22]. Sample items included: “A person can get HIV from oral sex,” and “There is a vaccine that can stop adults from getting HIV.” Response options included “yes, “no,” and don’t know.” HIV knowledge was scored such that a correct answer received a “1” and an incorrect answer received a “0”. Scores were summed, with higher scores indicating greater HIV knowledge.

PrEP awareness was assessed with the question “Have you ever heard of a daily pill that an HIV-negative person can take to prevent getting HIV prior to the survey?” and the response options were “yes”, “no”, or “I don’t know” Participants who responded “yes” were coded positive for PrEP aware.

### Binge drinking

Binge drinking, which has been shown to influence HIV prevention and PrEP use, increased sexual risk behaviors, and lower engagement in the PrEP care continuum [16] was included as a covariate in this study. Binge drinking was measured using a single item:“ Over the past month when you drank alcohol, what was the most number of drinks you drank on any one occasion?” Consistent with the definition of binge drinking for women used by the United States Abuse and Mental Health Services, participants who reported four or more drinks on any drinking occasion in the past month were coded positive for binge drinking [23].

### PrEP stigma

PrEP stigma was measured using a subset of 15 items from the original 22-item PrEP stigma scale, previously validated in the U.S. by Klein and Washington [24]. We selected 15 items based on their relevance to our target population and the revised scale (Cronbach’s α = 0.93). Sample item included: “People will think I am HIV-positive if I take PrEP.” Response options range from “strongly disagree” (1) to “strongly agree” (5). Scores were summed with higher scores indicating greater stigma.

#### HIV testing, perceived HIV risk, and worrying about HIV.

HIV testing in the past year was assessed by asking participants how often they get tested for HIV. Participants who responded that they had tested in one year or less were coded as “yes” for testing in the past year. Participants who responded as having tested more than a year or never tested were categorized as “No” for not testing in the past year. Perceived HIV risk was assessed by asking participants: “How likely is it that you will become HIV-positive in your lifetime?” Response options included “very unlikely,” “unlikely,” “somewhat likely,” “likely,” and “very likely.” Perceived HIV risk was dichotomized as “No” for those who responded as “very unlikely or unlikely” and “Yes” for those who responded as “very likely, likely, or somewhat likely.” STI history was assessed by asking participants if they had ever received an STI diagnosis from a clinician. Responses were coded as “yes/no.” Worrying about getting HIV was assessed by asking participants, “How often do you worry that you might get HIV?” Responses were “never/sometimes/often/always.” We dichotomized it to “yes/no” with yes (sometimes, often, and always).

#### Primary care clinician, health insurance, medical trust, healthcare stereotype, and distance to clinic.

Having a primary care clinician was assessed by asking participants if they have a regular doctor they visit (yes/no). Health insurance was assessed by asking participants if they had health insurance. Responses were recorded as “yes/no.” Medical trust was measured using a 6-item scale assessing trust in healthcare clinicians. We modified this scale from a 12-item group-based medical mistrust scale [25]. Example items: “My medical clinician makes me feel respected during medical appointments,” “I trust my medical provider.” Responses were based on a Likert-type scale ranging from 1 (strongly disagree) to 5 (strongly agree), with higher scores indicating greater trust. Reliability in the current sample was (Cronbach’s alpha = 0.73), which is above the accepted threshold. Healthcare stereotype threat was assessed using a modified, four-item scale by Abdou and Fingerhut [26]. Sample questions included:” When seeking healthcare, I worry about being negatively judged because of my race.” Response options range from “strongly disagree” (1) to “strongly agree” (4). Average scores were calculated for each participant, with higher scores indicating a greater level of healthcare stereotype threat. Cronbach’s alpha was 0.90 for this sample. The distance to the clinical clinician was assessed by asking participants, “How far away is your current medical clinician from where you live?” Responses were dichotomized ≤5 miles and > 5 miles.

### Statistical analysis

We describe the characteristics of the sample using means and standard deviations for continuous variables and frequencies and percentages for categorical variables. Sample characteristics were stratified by willingness to use LAI-PrEP (Table 1). We used Bivariate and Multivariate logistic regression models to assess the association between covariates and willingness to use LAI-PrEP. At the bivariate analyses, T-tests and Chi-square were used to compare continuous and categorical variables. Theoretically relevant variables (education and relationship status) and covariates that were significant at p < 0.05 in bivariate analysis were included in the multivariate logistic regression model for willingness to use LAI-PrEP. We evaluated estimates for statistical significance based on 95% confidence intervals using p < 0.05. Statistical analyses were performed using STATA version 18.

**Table 1:** Sample characteristics stratified by willingness to use LAI-PrEP (N = 491).

Variables	Total	Willingness to use LAI-PrEP		
	N = 491	Yes (n = 180)	No (n = 311)	P-value
Age (M(SD)	40.4 (17.5)	38.4 (16.8)	41.6 (17.8)	0.05
Education				0.84
<College	259 (52.8)	96 (53.3)	163 (52.4)	
≥College	232 (47.2)	84 (46.7)	148 (47.6)	
Income				0.11
<$12000	115 (23.4)	35 (19.4)	80 (25.7)	
≥$12000	376 (76.6)	145 (80.6)	231 (74.3)	
Relationship status				0.14
Single	389 (79.2)	149 (82.8)	240 (77.2)	
Partnered	102 (20.8)	31 (17.2)	71 (22.8)	
Distance to clinic				
≥5 miles	362 (73.3)	128 (71.1)	234 (75.2)	0.31
<5miles	129 (26.3)	52 (28.9)	77 24.7)	
Nativity				0.61
Foreign born	19 (3.9)	8 (4.4)	11 (3.5)	
US-Born	472 (96.1)	172 (95.6)	300 (96.5)	
Binge drinking				0.003
No	377 (76.8)	125 (69.4)	252 (81.0)	
Yes	114 (23.2)	55 (30.6)	59 (19.0)	
HIV Knowledge (M(SD)	5.9 (2.5)	5.9 (2.4)	5.9 (2.6)	0.63
PrEP stigma M (SD)	41.8 (12.7)	41.3 (13.1)	42.1 (12.5)	0.53
Healthcare stereotype M(SD)	9.9 (4.3)	9.4 (4.6)	10.2 (4.1)	0.03
HIV test past year				<0.001
No	319 (65.0)	96 (53.3)	223 (71.7)	
Yes	172 (35.0)	84 (46.7)	88 (28.3)	
Perceived HIV risk				0.19
No	384 (78.2)	135 (75.0)	249 (80.1)	
Yes	107 (21.2)	45 (25.0)	62 (19.9)	
Oral PrEP aware				<0.001
No	297 (60.5)	83 (46.1)	214 (68.8)	
Yes	194 (39.5)	97 (53.9)	97 (31.2)	
Any history of STIs				0.16
No	467 (95.1)	168 (93.3)	299 (96.1)	
Yes	24 (4.9)	12 (6.7)	12 (3.9)	
Primary care Clinician				<0.001
No	124 (25.3)	26 (14.4)	98 (31.5)	
Yes	367 (74.7)	154 (85.6)	213 (68.5)	
Health Insurance				0.17
No	63 (12.8)	28 (15.3)	35 (11.2)	
Yes	428 (87.2)	152 (84.4)	276 (88.8)	
Injectable contraceptives				0.16
No	427 (87.2)	151 (84.4	276 (88.7)	
Yes	63 (12.8)	28 (15.6)	35 (11.3)	
Interest in Oral-PrEP				<0.001
No	365 (74.3)	106 (58.9)	259 (83.3)	
Yes	126 (25.7)	74 (41.1)	52(16.7)	
Ever worry about HIV				<0.001
No	359 (73.1)	114 (63.3)	245 (78.8)	
Yes	132 (26.9)	66 (36.7)	66 (21.2)	
Medical trust (M ± SD)	19.1 (4.1)	19.7 (4.2)	18.6 (3.9)	<0.001

## Results

### Sample characteristics

Table 1 shows sample characteristics stratified by willingness to use LAI-PrEP. A total of 491 participants were included in this study. The mean age was 40.4 [standard deviation (SD)=17.5] years, many were above federal poverty levels (≥$12,000), and the majority (79%) were single. Most participants were U.S.-born. (94%), resided in urban/suburban settings of the U.S. South (80%). These states were Alabama, Arkansas, Delaware, the District of Columbia, Florida, Georgia, Kentucky, Louisiana, Maryland, Mississippi, North Carolina, Oklahoma, South Carolina, Tennessee, Texas, Virginia, and West Virginia. More than half (53%) had less than a college degree. Most participants reported that the distance to the clinic was five or more miles from where they live (73.3%). Overall, a small proportion of participants perceived themselves to be at risk of getting HIV (21.3%), more than one-third reported having been tested for HIV in the past 12 months (35%), and about 5% reported a history of STI diagnosis. Most participants had a regular primary care clinician (74.7%) and health insurance (87.2%).

Mean HIV knowledge was 5.9(SD = 2.5, range from 0–10). Medical mistrust was 19.1 (SD = 4.1) on a range from 6–29. In bivariate analyses (Table 1), participants who intended to use LAI-PrEP were slightly younger (Mean age 38.4 vs 41.6 years, p = 0.05), were more likely to have binge drank alcohol in the last month (30.6% vs 19.0%,p = 0.003), were more likely to have tested for HIV in the past year (46.7% vs 28.3%, p < 0.001), were aware of PrEP (53.9% vs 31.2%), p < 0.001), had a primary care clinician (85.6% vs 68.5%, p < 0.001), worried about HIV (36.7% vs 21.2%, p = 0.001), and reported higher levels of trust in the medical care system [Mean score 19.7 (SD = 4.2) vs 18.6 (SD = 3.9), p < 0.001]. However, those with higher scores on healthcare stereotype threat were less likely to want to use LAI-PrEP [Mean score 9.4 (SD = 4.6) vs. 10.2 (SD = 4.1), p = 0.03].

### Factors associated with LAI willingness

Approximately 40% of participants were aware of PrEP, and 36.7% (n = 180) indicated a willingness to use LAI-PrEP. In the adjusted model (Table 2), four factors were independently associated with the willingness to use LAI-PrEP. Participants who were aware of oral PrEP prior to this study were more than twice as likely to express a willingness to use LAI-PrEP compared to those who were not aware of PrEP (aOR=2.37, 95% CI = 1.50, 3.73, P < 0.001). Similarly, individuals who reported having a regular clinician were also twice as likely to intend to use LAI-PrEP (aOR=2.01, 95% CI = 1.10–3.68, P = 0.02). Other factors statistically significantly associated with higher odds for willingness to use LAI-PrEP included worry about HIV (aOR=1.78, 95% CI = 1.09, 2.89, P = 0.02) and trust in medical care system (aOR=1.41, 95% CI = 1.03, 1.93, P = 0.03). In contrast, negative perceptions and stereotypes about healthcare were significantly associated with lower odds of being willing to use LAI-PrEP (aOR=0.94, 95% CI = 0.89–0.99, P = 0.04).

**Table 2:** Multivariate logistic regression of factors associated with willingness to use LAI-PrEP (N = 491).

Variables	Willingness to use LAI-PrEP			
	Unadjusted odds ratio [95% CI]	P values	Adjusted odds ratio [95% CI]	P-value
Age	0.98 (0.97, 0.99)	0.05	0.99 (0.97, 1.01)	0.31
Education level				
≥College	0.96 (0.67, 1.39)	0.84	0.69 (0.44, 1.01)	0.12
<College (ref)				
Relationship status				
Married	0.70 (0.44, 1.12)	0.14	0.71 (0.41, 1.22)	0.21
Single (ref)				
Income levels				
≥ $12,000	1.43 (0.92, 2.25)	0.11	1.46 (0.83, 2.59)	0.19
<$12,000 (ref)				
Binge drinking				
Yes	1.87 (1.23, 2.88)	0.004	1.30 (0.77, 2.18)	0.32
No (ref)				
PrEP aware				
Yes	2.58 (1.76, 3.76)	<0.001	2.37 (1.50, 3.73)	<0.001
No (ref)				
Has clinician				
Yes	2.73 (1.68, 4.40)	<0.001	2.01 (1.10, 3.68)	0.02
No (ref)				
HIV worried				
Yes	1.90 (1.25, 2.91)	0.003	1.78 (1.09, 2.89)	0.02
No (ref)				
HIV testing past 12 months				
Yes	1.60 (1.09, 2.34)	0.01	1.24 (0.77, 1.96)	0.36
No (ref)				
Medical trust	1.84 (1.39, 2.43)	<0.001	1.41 (1.03, 1.93)	0.03
Healthcare stereotype	0.95 (0.91, 0.97)	0.03	0.94 (0.89, 0.99)	0.04

Covariates adjusted in logistic regression model include: Age, education, relationship status, binge drinking, PrEP awareness, HIV testing in the past 12 months, Worrying about HIV, Having a clinician, medical trust, and healthcare stereotype.

## Discussion

This study investigated factors associated with the willingness to use LAI-PrEP among Black cisgender women residing in the U.S. South who would be eligible for PrEP. We found that awareness of PrEP, having a regular clinician, concern about HIV, and trust in medical care were associated with the willingness to use LAI-PrEP. However, negative stereotypes about healthcare were significantly associated with lower odds of willingness to use LAI-PrEP. These results provide important insights into the complex interplay of factors influencing LAI-PrEP willingness in this population.

The positive association between PrEP awareness and willingness to use LAI-PrEP aligns with previous studies, which demonstrate that awareness is an important first step in PrEP initiation [27–30]. A systematic review by Mayer et al.(2020) showed that PrEP awareness was significantly associated with PrEP uptake [27]. Similarly, Biello et al. (2018) found that among men who have sex with men (MSM) in the U.S., those who were aware of daily oral PrEP were more likely to express interest in using LAI-PrEP [28]. A qualitative study by Philbin et al. (2021) among women in the U.S. found that women who were aware of PrEP in general expressed high interest in LAI-PrEP [29]. It is worth noting that most of these studies were conducted among MSM, with only one qualitative study among women [29]. Studies with women were mostly conducted with transgender women, qualitative, conducted in U.S. urban areas, and mostly among young people. Our study provides novel insights into the perspectives of cisgender Black women, with a mean age of 41 years. This is particularly important given the underrepresentation of Black women in previous PrEP research and the high HIV burden in the Southern U.S. While the CDC latest data indicate that young MSM experience the highest HIV incidence, Black women’s rates remain relatively consistent across age groups [2]. Interventions to increase PrEP uptake should incorporate strategies tailored explicitly to Black women throughout their lifespan, including those in their late reproductive years and beyond. A notable finding from our study is the potential appeal of LAI-PrEP among young women. Given the growing evidence supporting the efficacy of injectable PrEP [31], there is a need to expand its availability in formats that appeal to young people. Future research should explore interventions to facilitate LAI-PrEP access and adherence among young populations of women at risk of HIV.

The significance of having a clinician in predicting LAI-PrEP willingness highlights the role of healthcare access in HIV prevention. This finding is particularly relevant in the context of the U.S. South, where healthcare disparities are pronounced, and many individuals lack access to regular medical care [32]. As of 2024, several Southern states still have not expanded Medicaid under the Affordable Care Act (ACA), despite the potential benefits for their uninsured populations [33]. Medicaid is the largest public health insurance coverage for people with HIV in the U.S., and its role continues to expand, especially for low-income individuals [33]. Our results are consistent with previous studies, which have shown that having a regular clinician facilitates ongoing engagement in care [28,29,34]. For instance, Philbin et al. found that women who had regular clinicians reported more interest in LAI-PrEP [29]. Biello et al. (2018) noted that regular contact with clinicians facilitated ongoing PrEP education and support for people who inject drugs [28]. In another study among young Black MSM, those with a primary care clinician were more likely to express interest in PrEP. However, the study noted challenges in translating interest into actual uptake, highlighting the need for clinician education and support [34]. Another study, although not specifically about LAI-PrEP, found that MSM in Ghana who had a regular clinician were more likely to be aware of and interested in PrEP [35]. These findings collectively suggest that expanding access to primary care, especially in vulnerable communities, could improve the reach of LAI-PrEP. Consistent engagement with the healthcare system may increase opportunities for clinicians to recommend LAI-PrEP and for patients to feel supported in their decision-making processes. Thus, expanding insurance coverage and access to primary care, particularly in marginalized communities, could improve the reach of LAI-PrEP.

Our findings demonstrating that concern about HIV was a significant predictor of LAI-PrEP willingness suggest that risk perception plays a crucial role in motivating preventive behaviors. This aligns with prior studies such as Levy et al. [28], which found that MSM with higher perceived HIV risk and recent STI diagnosis were more willing to use LAI-PrEP. In another study by Holloway et al. (2017), which focused on young MSM, higher HIV worry was associated with greater PrEP uptake. However, it is important to note that previous studies have found discrepancies between actual HIV risk and perceived risk among Black women [36]. Future interventions should focus on accurately communicating HIV risk while using non-stigmatizing language. Future public health messaging should address these concerns while providing information on LAI-PrEP efficacy to encourage its adoption.

The positive correlation between trust in medical care and willingness to use LAI-PrEP among Black women in the U.S. South is an encouraging finding, especially given the historical medical mistrust among Black communities in the U.S. [19,37,38]. This relationship suggests that initiatives aimed at promoting trust between clinicians and Black women can yield concrete benefits for HIV prevention efforts. Healthcare systems and clinicians should prioritize cultural competence, transparency, and community engagement as strategies to build trust and improve health outcomes [39]. This trust-building process will be instrumental in increasing LAI-PrEP uptake and, more broadly, in enhancing engagement with HIV prevention services [40,41]. However, negative healthcare stereotypes still hinder LAI-PrEP uptake, reflecting broader issues of medical mistrust among Black communities [38,42]. The intersectionality of race, gender, and geographic location in our study population cannot be overstated. Black women in the Southern US face unique challenges related to HIV prevention, including structural racism, gender-based discrimination, and regional healthcare disparities [43,44]. Addressing these issues requires tailored, culturally appropriate interventions that consider the historical underrepresentation of Black women in PrEP research and campaigns [45,46]. Overcoming barriers to LAI-PrEP adoption necessitates culturally competent care, community-based approaches, and efforts to address healthcare inequities [12].

This study contributes to the growing body of literature on the factors influencing PrEP uptake, particularly for the emerging LAI-PrEP option. However, there are limitations to note. First, our focus on willingness rather than actual use of LAI-PrEP means that there may be additional barriers between willingness and action that were not captured in this study. Second, at the time of surveying participants, oral PrEP was a widely known form of PrEP; thus, PrEP awareness in the survey focused on daily oral pills, while the willingness measured was for LAI-PrEP. This difference in PrEP form could influence participant responses, as their awareness of oral PrEP might not fully translate to willingness to use LAI-PrEP. This limitation may have led us to over- or under-estimate the relationship between PrEP awareness and LAI-PrEP willingness. Future research should consider measuring awareness of oral PrEP, LAI-PrEP, and other emerging PrEP formulations to avoid this bias. Third, the cross-sectional nature of our data does not allow us to establish causality between the identified factors and LAI-PrEP willingness. Longitudinal studies are needed to understand better how these factors influence LAI-PrEP uptake over time. Also, recall bias, participants were asked about HIV testing in the past year, and the last time they had a doctor’s check-up. Although the online survey format may reduce social desirability bias, some participants may still have reported a willingness to use PrEP when they do not intend to do so. Finally, while our study focused on the U.S. South, regional variations within the South were not explored and could be a critical area for future research.

Despite these limitations, our findings have important implications for public health practice and policy. They suggest that a multifaceted approach is needed to promote LAI-PrEP uptake among Black cisgender women in the South. Study results suggest that increasing awareness of PrEP, particularly for cisgender Black women (>40years), fostering positive relationships with clinicians, addressing HIV-related concerns, and building medical trust are critical strategies for promoting the use of LAI-PrEP. At the same time, healthcare systems need to actively work to mitigate negative stereotypes and rebuild trust, particularly among populations that have historically faced discrimination and inadequate care. Future research should explore targeted interventions that address these factors to optimize the uptake of LAI-PrEP and other HIV prevention strategies in high-risk communities.

In conclusion, our study provides valuable insights into the factors influencing LAI-PrEP willingness among Black cisgender women in the U.S. South. The findings suggest that a combination of behavioral, structural barriers, such as healthcare access, and psychosocial factors, including trust in medical care, shape the willingness to use LAI-PrEP. Interventions aimed at increasing LAI-PrEP uptake should focus on promoting HIV testing, enhancing patient-clinician communication, addressing healthcare stereotypes, and improving PrEP awareness, particularly among individuals who are at increased vulnerability to HIV. Tailored strategies that account for the unique social and cultural contexts of Black cisgender women in the Southern U.S. are essential to closing gaps in PrEP utilization and reducing HIV disparities.
